# The Developmental Basis of Quantitative Craniofacial Variation in Humans and Mice

**DOI:** 10.1007/s11692-012-9210-7

**Published:** 2012-11-20

**Authors:** Neus Martínez-Abadías, Philipp Mitteroecker, Trish E. Parsons, Mireia Esparza, Torstein Sjøvold, Campbell Rolian, Joan T. Richtsmeier, Benedikt Hallgrímsson

**Affiliations:** 1 Department of Anthropology, Pennsylvania State University, University Park, PA USA; 2 Department of Theoretical Biology, University of Vienna, Vienna, Austria; 3 Department of Cell Biology and Anatomy, Faculty of Medicine, McCaig Institute for Bone and Joint Research, Alberta Children’s Hospital Research Institute, University of Calgary, Calgary, Canada; 4 Department de Biologia Animal, Secció d’Antropologia, Universitat de Barcelona, Barcelona, Spain; 5 Osteologiska enheten, Stockholms Universitet, Stockholm, Sweden; 6 Present Address: CRG, Center for Genomic Regulation, Dr. Aiguader, 88, 08003 Barcelona, Spain

**Keywords:** Mouse, Human: Geometric morphometrics, Craniofacial morphology, Morphological integration, Evolution and development

## Abstract

The human skull is a complex and highly integrated structure that has long held the fascination of anthropologists and evolutionary biologists. Recent studies of the genetics of craniofacial variation reveal a very complex and multifactorial picture. These findings contrast with older ideas that posit much simpler developmental bases for variation in cranial morphology such as the growth of the brain or the growth of the chondrocranium relative to the dermatocranium. Such processes have been shown to have major effects on cranial morphology in mice. It is not known, however, whether they are relevant to explaining normal phenotypic variation in humans. To answer this question, we obtained vectors of shape change from mutant mouse models in which the developmental basis for the craniofacial phenotype is known to varying degrees, and compared these to a homologous dataset constructed from human crania obtained from a single population with a known genealogy. Our results show that the shape vectors associated with perturbations to chondrocranial growth, brain growth, and body size in mice do largely correspond to axes of covariation in humans. This finding supports the view that the developmental basis for craniofacial variation funnels down to a relatively small number of key developmental processes that are similar across mice and humans. Understanding these processes and how they influence craniofacial shape provides fundamental insights into the developmental basis for evolutionary change in the human skull as well as the developmental-genetic basis for normal phenotypic variation in craniofacial form.

## Introduction

Animal models are fundamental to the study of the developmental basis for human birth defects. It is thus well accepted that inferences can reliably be made from animal models such as the mouse or chick to reveal the developmental basis for phenotypic extremes represented by disease and conditions such as cleft lip (Juriloff and Harris 2008) or holoprosencephaly (Young et al. 2010). Interestingly, the proposition that similar inferences can be made about the developmental basis for smaller scale, non-pathological variation in humans is less straightforward. It is not a given that variation in homologous developmental processes, such as the expression of a particular gene, produces homologous variation at the phenotypic level in mice and humans, for example. We assume that this is normally the case and that this assumption is more valid the more closely related the species being compared are. In fact, the assumption that similar processes and genetic mechanisms underlie the phenotypic range in mice and humans underlies the body of work on the genetics and genomics of complex traits in mice (Churchill et al. 2004; Williams et al. 2004; Aylor et al. 2011; Iraqi et al. 2012), including the shape of the mouse cranium (Leamy et al. 1999; Mezey et al. 2000; Klingenberg et al. 2001). The validity of such inferences is an important issue for the evolutionary developmental biology of humans. Understanding the developmental basis for the generation of phenotypic variation is the key to unraveling the complex interplay of development and evolution in human evolution (Raff 1996; Lieberman et al. 2004; Lieberman 2010) and this can only be studied in animal models such as mice. The difficulty, however, is that we really do not know to what extent—at a quantitative level—the phenotypic effects of genetic variation in mice resemble that of human genetic variation. This study is a limited and targeted effort at addressing this important question for human evolutionary developmental biology.

Studies of the genetics of normal phenotypic variation in humans reveal an extraordinarily complex picture. For human height, for example, genome-wide association studies of very large cohorts reveal very many loci of small effect (Weedon et al. 2008) and multivariate approaches to similar datasets show that a large proportion of the variance in human height is due to many common variants with very small effects (Yang et al. 2010). Large-scale GWAS studies for craniofacial shape are underway for humans, but it is likely that many studies of complex phenotypic traits will reveal similar results (Gibson 2010). This picture of the genetic architecture of complex phenotypic traits makes it extremely unlikely that a specific gene-focused approach will reveal significant insights into the developmental basis for craniofacial evolution in humans. Instead, conceptual approaches that abstract or simplify complexity at the genetic level are essential to making meaningful inroads into the evolutionary-developmental biology of most complex phenotypic features including the human craniofacial complex (Hallgrímsson and Lieberman 2008).

One such paradigm is the “middle-out” approach which focuses on high-level development processes and their phenotypic effects (Hallgrímsson and Lieberman 2008; Hallgrímsson et al. 2009; Hallgrímsson and Hall 2011). This paradigm maintains that despite the complex genetic organization in mammals, numerous genes and developmental processes often ‘‘funnel’’ down to a few processes and properties that determine the adult phenotype. In other words, the effects of multiple genes are mediated via the same pathway or developmental process. For example, many genes are involved in cranial development but it is likely that the overall cranial shape is basically influenced by a few factors, such as brain size relative to facial size (Hallgrímsson et al. 2007a; Bastir et al. 2010). In a statistical language (e.g., Pearl 2000), the variable brain size would “screen off” all the genes affecting brain size from cranial shape: many genes and developmental processes contribute to variation in brain size, which in turn affects cranial shape, but conditioning on (observing) brain size renders cranial shape statistically independent from all these genes. In other words, when knowing brain size, learning about all the underlying genes does not contribute to our knowledge of cranial shape (at a statistical level). Importantly, the functional consequences of exactly how and why brain size is changing are, of course, under selection for reasons that have to do with the function of the brain. So we are not arguing that genes that influence brain size in different ways are screened off from selection. Rather, the functional effects of genes that influence brain size and the function of the brain are not relevant to explaining how those genes influence craniofacial shape, because those effects are funneled through the much simpler interactions between the growth of the brain and the shape of the skull.

If this is true, then modeling the impact of brain size on cranial shape would significantly contribute to our understanding of the development of cranial shape—whatever the actual genetic basis for variation in brain size might be. One approach to investigate the nature of this effect is to examine the statistical relationship between brain size and craniofacial shape in mutant mice with enlarged brains (Hallgrímsson and Lieberman 2008). Such a statistical model needs to be validated, however, because we do not know to what extent the cranial shape changes that occur as the result of enlarged brains in mice relate to a homologous set of changes that would occur in the human cranium as a result of the same perturbation.

The study of integration is crucial to unraveling the developmental basis for phenotypic variation and evolutionary change (Ross and Ravosa 1993; Lieberman et al. 2000; González-José et al. 2004; Mitteroecker and Bookstein 2008; Bastir et al. 2010). Integration, or the *tendency* for structures to covary, is determined by developmental connections between traits such as influence from common developmental processes or the indirect effects of parallel developmental or environmental effects (Klingenberg 2005). Importantly, patterns of covariation are produced when there is underlying variation in the processes that produce these direct or indirect connections among traits (Mitteroecker and Bookstein 2007; Hallgrímsson et al. 2007b, 2009; Mitteroecker 2009). Developmental processes that funnel or screen-off genetic variation often are central to complex developmental interactions and hence tend to result in major patterns of phenotypic covariation in a complex trait. This concept is related but different from the idea of buffering or canalization (Waddington 1957). Canalization refers to the compensation of perturbations in the course of development and hence to the production of a stable adult phenotype despite environmental or genetic variation. Funneling, by contrast, is an overtly hierarchical view of development in which processes acting at some levels are more relevant to explaining the end-point phenotypic variation than others. Which levels are most relevant depends on the particular developmental context.

In humans, studies of integration patterns are limited by the fact that the underlying developmental sources of variation cannot be controlled. Because there are many processes that produce phenotypic covariation, this produces a “palimpsest effect” where it is often difficult or even impossible to interpret the developmental story that corresponds to a pattern of phenotypic covariation (Hallgrímsson et al. 2007b). Animal models represent the key to this problem because variation can be introduced in ways that are at least in large part understood for particular model systems. A gene effect that perturbs a particular developmental process, such as neural crest migration into the face, for example, can become an assay to determine the phenotypic consequences of that process. Importantly, such studies tell us virtually nothing about the role of the particular gene perturbed in normal phenotypic variation because of the way that developmental processes “screen off” the effects of multiple genes.

Despite the palimpsest problem, studies of phenotypic covariation patterns have revealed substantial insight into the architecture of craniofacial variation in humans. Most importantly, these studies reveal the extent to which the craniofacial complex is highly integrated, even at the genetic level (Mitteroecker and Bookstein 2008; Mitteroecker et al. 2012; Martínez-Abadías et al. 2012). By this we mean that the total phenotypic variance is captured by relatively few factors when subjected to multivariate reduction techniques such as principal components analysis (Martínez-Abadías et al. 2012). These results are encouraging for use of the “middle-out” approach, because they suggest a tractable level of complexity for the major developmental processes that structure the variation of the craniofacial complex. At the very least, they suggest that major features of the structure of variation in this developmentally and genetically complex structure can be related back to identifiable developmental processes.

In previous work, we have shown that large-scale developmental processes such as brain growth, facial prominence outgrowth, chondrocranial growth and overall body growth have large and predictable effects on the overall shape of mouse skull (Hallgrímsson 1993; Lieberman et al. 2008; Hallgrímsson and Lieberman 2008; Hallgrímsson et al. 2007a, 2009). Martínez-Abadías and collaborators have shown that the genetic and phenotypic integration patterns of the skull are fairly congruent and correspond to the three major developmental/functional regions of the skull (face, neurocranium and basicranium) (Martínez-Abadías et al. 2009a, 2009). In this study, we build on this combined body of work to investigate whether these integrated axes of variation in mice correspond to features of phenotypic variation in humans. Specifically, we study the effect of brain size, chondrocranial length, and overall size on cranial shape both in mice and humans. We validate these models in mice by relating the estimated relationships to a range of mutations with known effects. Comparing the statistical patterns in mice and humans allows us to estimate to which degree homologous developmental processes in mice and in humans produce quantitatively similar patterns of phenotypic variation. This is important for the use of mice to study the developmental basis for phenotypic variation in the human cranium.

## Materials and Methods

Our mouse sample consisted of 156 adult crania from 7 inbred strains of different genetic backgrounds, which included mice with wildtype phenotype (C57Bl/6J*Balb6, *Balb/c*, and *C57Bl/6J*) and mice with altered skull morphology due to mutations that affect the growth of the brain (*Mceph/Mceph*), the chondrocranium (*Papps2*-*/Papps2*-) and overall size (*ghrhr*-/-) (for more details see Table 1). To assess if the phenotypic effects of each of these genetic mutations in mouse models correspond to axes of morphological variation in modern humans, we analyzed a sample of 390 adult human skulls from Hallstatt (Austria) (Martínez-Abadías et al. 2009a, 2012).

**Table 1:** Pairs of wildtype/mutant mouse strains used for each analysis

Analysis	Strain	Genotype	Phenotype	N
Brain	C57Bl/6J*Balb6	Wildtype	None	14
		*Mceph/Mceph*	25–30 % increase in brain size	18
Chondrocranium	*C57Bl/6J*	Wildtype	None	48
		*Papps2/Papps2*-	Reduction of the chondrocranium, shortening of the face, and doming of the neurocranium	26
Overall size	*C57Bl/6J*	Wildtype	None	48
		*ghrhr*-/-	Reduced body size	11

Sample sizes (*N*) are provided in the last column. For more detailed information on the phenotype and the effect of the genetic mutations see Hallgrímsson et al. (2004, 2007a, 2009)

**Fig. 1:**
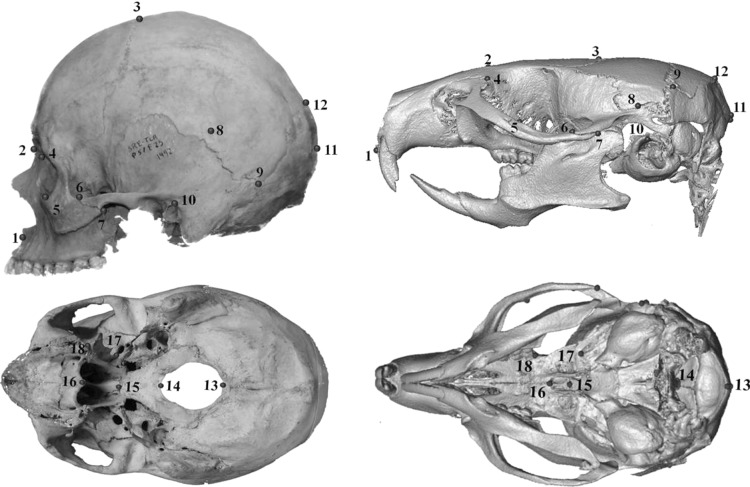
The set of 3D homologous landmarks used to compare human and mouse crania

Three-dimensional (3D) coordinates of a set of 18 homologous landmarks from the left side of mice and human skulls (Fig. 1) were recorded to allow comparison between mouse and human datasets. Mouse crania were micro CT-scanned (Scanco Viva-CT40) at 35 μm resolution (70 kV, 160 μA, 500 projections) and landmarks were digitized on the 3D isosurfaces reconstructed using Avizo 6.0 (Visualization Sciences Group, VSG). Landmark coordinates were recorded on human crania using a Microscribe G2X digitizer (Immmersion, Inc.).

### Shape Analysis

Geometric morphometric (GM) methods were used to explore skull shape variation and to estimate the effects on skull shape of brain size, chondrocranial length and overall size in mice and humans. GM is based on Generalized Procrustes Analysis (Dryden and Mardia 1998; Rohlf and Slice 1990), a least-squares oriented procedure that extracts shape information from the raw landmark coordinates by standardizing for scale, position, and orientation of the landmark configurations. Centroid size, computed as the square root of the summed squared distances between each landmark and the centroid of the landmark configuration (Dryden and Mardia 1998), is used as a measure of scale.

We estimated brain size, chondrocranial length and overall size both in the mouse and the human sample, and used these measurements as predictor variables in our shape analysis. Mouse brain size was measured as the endocranial volume of virtual endocasts obtained after image analyses. Image datasets were first subjected to median and maximum filtering in ImageJ to automatically remove pseudoforamina and sutures, and the final virtual endocasts were created after edition in Analyze 3D 5.0 to remove the brain stem and any non-endocranial projections. Human brain size was measured as the cranial capacity of dry crania filled with seeds. All brain volumes were converted to their cube root. Chondrocranial length was estimated as the linear distance between the landmarks hormion and opisthocranion; and overall size was computed as the log centroid size of the whole set of cranial landmarks.

### Mouse Models

We examined the average skull shape changes induced by the mutations that alter the size of the brain, chondrocranium, and overall skull by contrasting the three mutant mouse models with a wildtype strain of the same genetic background.

1. To analyze the effect of brain size on skull shape, we used the mutant *Mceph/Mceph* homozygous mouse model (the Jackson Laboratory), which carries a recessive autosomal 11-bp deletion in the Knca1 gene (Diez et al. 2003; Petersson et al. 2003) that generates a 25–30 % expansion of brain size due to generalized neural cell hypertrophy. *Mceph/Mceph* mice were compared to a sample of wildtype mice with mixed C57Bl/6J*Balbc/ByJ background.
2. To analyze the effect of chondrocranial size, we used the Brachymorph mouse model (the Jackson Laboratory), an inbred mice strain with *C57Bl/6J* background that carries a mutation that affects chondrocranial development by reducing its growth. Brachymorph mutant mice are homozygotes of an autosomal recessive mutation in the phosphoadenosine–phosphosulfate synthetase 2 gene (*Papps2*-*/Papps2*-) and have shorter cranial base because the genetic mutation reduces chondrocranial growth via undersulfation of glycosaminoglycans in cartilage matrix (ul Haque et al. 1998, Kurima et al. 1998). Brachymorph *Papps2*-*/Papps2*- mice were compared with wildtype *C57Bl/6J* mice.
3. To assess the effect of overall skull size, we used mice with a null mutation in the growth hormone releasing hormone receptor (*ghrhr*-/-*)* (Godfrey et al. 1993) on the *C57BL/6J* background. These ‘little’ mice are dramatically reduced in body size due to deficient synthesis of growth hormone. The difference in shape between them and the wildtype strain is, therefore, an allometric consequence of overall reduction in growth because the mutation affects body size and not craniofacial development directly or exclusively. Little mice exhibit normal prenatal growth but dramatically reduced post-natal growth with adult sizes averaging less than 50 % of those attained by heterozygous littermates at 90 days (Gonzáles et al. in prep). Little *ghrhr*-/- mice were compared with wildtype *C57Bl/6J* mice.

### Comparing Normal Skull Shape Variation in Mice and Humans to Murine Mutations

The average effect of each genetic mutation was estimated by the difference between the average shape of the reference wildtype strain and the average shape of the mutated strain. The amount of this mutation effect was quantified as the Procrustes distance between the two average shapes, which is approximated by the Euclidean distance between the two sets of Procrustes shape coordinates (Rohlf 1999). The spatial pattern of the mutation effect was visualized by a thin-plate spline deformation grid between the two average shapes (Bookstein 1991).

We further explored the effects of brain size, chondrocranial length and overall size on normal (i.e. non-genetically disturbed) cranial shape variation using a sample of a wildtype mouse strain (*C57Bl/6J*) and a sample of modern humans. The effects were estimated by multivariate regressions of the skull shape (represented by the Procrustes shape coordinates) on the predictor variables (brain size, chondrocranium length and overall skull size). Separate multivariate regression analyses were computed for mice and humans. The regressions were pooled within sexes to account for sexual dimorphism, but individual scores along the resulting vector of regression coefficient were computed by projecting the original shape coordinates onto this vector. The multivariate regressions allowed us to determine normal skull shape patterns associated with small and large brains, chondrocraniums and skulls. These patterns were visualized using wireframes, 3D morphings and deformation grids. The statistical significance of mean differences and shape regressions was computed by permutation tests using 5,000 random permutations.

To determine if the skull shape changes resulting from the murine genetic mutations correspond to shape differences associated with normal variation of brain size, chondrocranial length and overall skull size in mice and humans, we compared the estimated average effect of each of the genetic mutations with the shape patterns obtained from the multivariate regressions. Similar changes in skull shape would indicate that mouse and human crania respond similarly to the influence of brain, chondrocranial and overall skull size. We related the spatial pattern of the mutation to the corresponding shape regression in mice and humans by visual comparison of the deformation grids. A quantitative comparison (e.g., by the angle between the shape vectors) is not directly interpretable because of the large mean shape differences between mice and humans. Even though all landmarks are biologically homologous in both species, landmark shifts may still not be comparable. For example, the foramen magnum is approximately horizontally oriented in humans and nearly vertically in mice (Fig. 1). A vector describing anterior–posterior variation in the landmarks of the foramen magnum thus would have very different biological implications in mice than in humans.

To quantify the contribution of the predictor variables to the observed shape variation, we calculated the fraction of total variance accounted for by these factors. A small fraction indicates a wide range of additional genetic and non-genetic factors influencing cranial shape, whereas a large fraction indicates that variation in cranial shape is due mainly to variation in the predictor variable. We further compared the variability of brain size, chondrocranial length and overall size between mice and humans. Because the mean values of these measures differed substantially between mice and humans, we compared the coefficients of variation (standard deviation divided by the mean) for all variables across the two species.

The observed statistical relationship between cranial shape and the predictor variables may not exclusively owe to direct causal effects of the predictor variables. Additional factors with causal effects on both cranial shape and the predictor variables can increase or decrease correlations due to the direct causal effects of the predictor variables. Likewise, multiple causal factors with opposite effects can lead to a low net correlation. For example, in the shape regression on brain size we assumed that changes in overall cranial shape are directly caused by brain size (which is of course an idealization; see the “Discussion” section), yet other genetic and/or epigenetic factors may influence cranial shape and brain size simultaneously and hence induce “spurious” correlation. Because we did not measure any of those additional factors, we had to estimate their effect in another than the usual path model approach (e.g., Pearl 2000). If we assume, for example, that in the *Mceph* mutation the change of cranial shape is caused only by the change of brain size, we can compare the amount of shape change induced by the observed amount of change in brain size to that observed in the regression study. If the amount of shape change per unit size change is the same in the mutation study and the regression study, no spurious correlation seems to affect the regression approach. Deviations would indicate the presence of additional factors increasing or decreasing the correlation caused by direct causal effects.

Statistical and morphometric analyses were performed in MorphoJ (Klingenberg 2011) and Mathematica 8.0.

## Results

### Brain Size

**Fig. 2:**
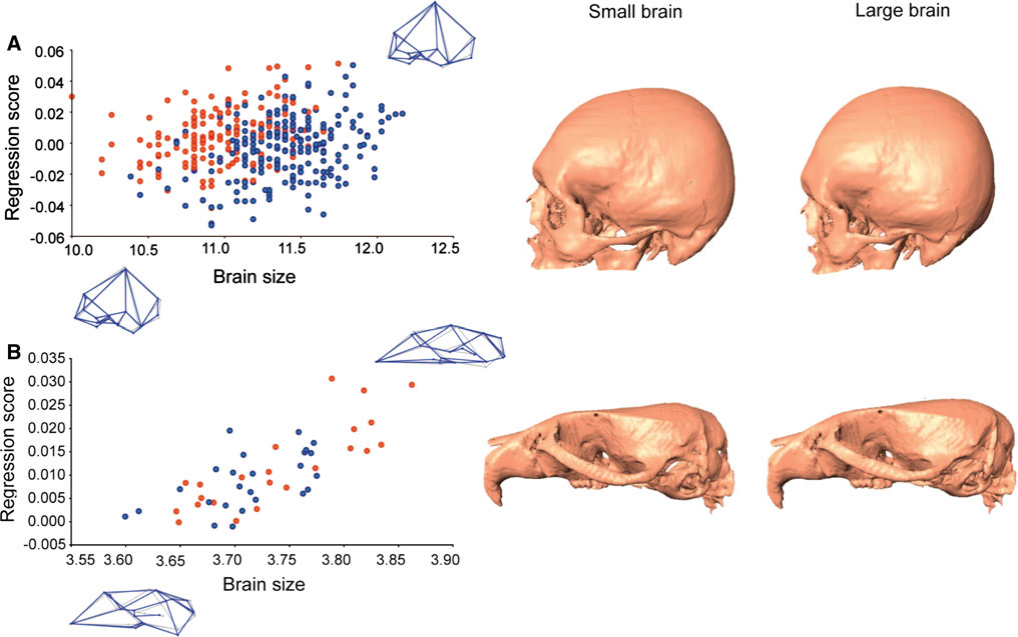
Multivariate regression of cranial shape on brain size in **a** modern humans and **b** wildtype *C57Bl/6J* mice. Regressions are pooled within sexes (*orange* females, *blue* males). Wireframes and 3D morphings show the morphology associated with low (*left*) and high (*right*) values of brain size (Color figure online)

The multivariate regression of hemicranial shape on brain size showed that in humans brain volume explains a low but statistically significant percentage of total morphological variation (1.35 %; *p* = 0.0001). In C57Bl/6J*Balb6 wildtype mice, the percentage of explained variation is higher than in humans (4.22 %; *p* = 0.0086), even though the human coefficient of variation for brain size is twice as large as for mice (0.102 vs. 0.048). Skull shape changes associated with small and large brains are similar in wildtype mice and humans (Fig. 2). In both species, an increase of brain size is associated with an expanded braincase: bregma moves upwards and forward increasing the height of the skull, euryon moves laterally widening the skull, and lambda and opisthocranion move outwards to a more postero–inferior position increasing the length (Fig. 2). In humans, these cranial vault changes are integrated with a retraction of the inferior face (subspinale and zygoorbitale move to a more postero–inferior position) and an increase in cranial base flexion (basion and opisthion move to a more antero–superior position, hormion moves upwards and alveolar point moves backwards) (Fig. 2a). The latter facial and cranial base changes only partially correspond to shape changes observed in mouse crania (Fig. 2b).

Statistical comparison of *Mceph/Mceph* mutant mice and C57Bl/6J*Balb6 wildtype mice showed that brain volume is 17 % larger in *Mceph/Mceph* mutant mice (*p* < 0.001), and that cranial shape difference between mutant and wildtype mice (as measured by the Procrustes distance) is 0.032 Procrustes units. We can predict the amount of cranial shape change that would result from a 17 % increase of brain volume in a normal population of mice using the multivariate regression in the *C57Bl/6J* wildtype mice sample. The predicted amount of shape change is equal to a Procrustes distance of 0.020, which is less than the cranial shape difference between *Mceph/Mceph* mutant mice and C57Bl/6J*Balb6 wildtype mice.

**Fig. 3:**
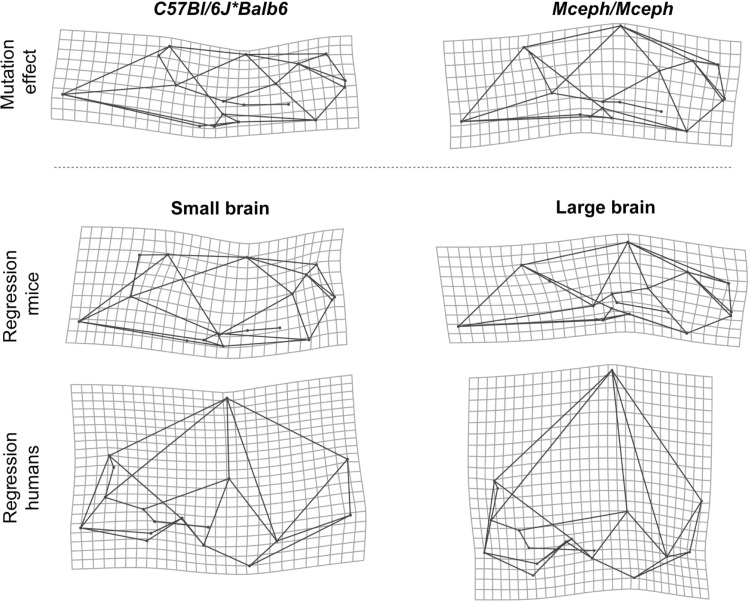
Morphological correspondence between the mutation effect on brain size and normal cranial shape variation in wildtype mice and humans. *Top row* average shape differences between C57Bl/6J*Balb6 wildtype mice and *Mceph/Mceph* mutant mice with increased brain size (*left* shape changes from mutant to wildtype, *right* shape changes from wildtype to mutant). *Middle and bottom rows* TPS grids from multivariate regression of cranial shape on brain size representing the cranial shape associated with small and large brains in *C57Bl/6J* mice and modern humans. Shape changes are exaggerated for visualization

Visual comparison of the cranial phenotypic effect of the *Mceph* mutation on brain size with normal skull variation associated with small and large brains (Fig. 3) indicates that there is a clear correspondence between the shape patterns. In comparison with C57Bl/6J*Balb6 wildtype mice, *Mceph/Mceph* mutant mice have larger brains and neurocrania with a relatively higher cranial vault, a flexed cranial base with shortened chondrocranial length, and a shorter and more vertically oriented face. Very similar shape patterns are observed in large-brained wildtype mice, and these patterns also correspond to the cranial shape of human specimens with larger brains (Fig. 3).

### Chondrocranial Size

**Fig. 4:**
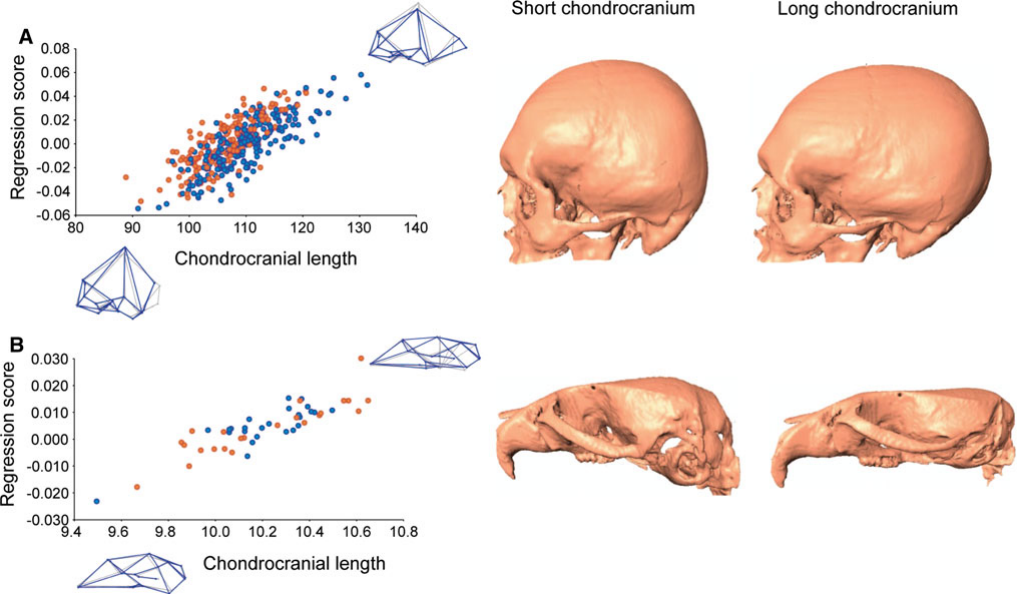
Multivariate regression of cranial shape on chondrocranial length in **a** modern humans and **b** wildtype *C57Bl/6J* mice. For more details see Fig. 2

Of the three morphological factors, chondrocranial length explained the highest percentage of total morphological variation in the human sample (7.28 %; *p* < 0.0001). In the *C57Bl/6J* wildtype mouse sample, chondrocranial length explained a percentage of total morphological variation halfway between brain size and overall cranial size (6.84 %; *p* < 0.0001). The coefficient of variation for chondrocranial size in mice is 0.024 and 0.059 in humans. Skull shape changes associated with longer/shorter chondrocranial length are similar in wildtype mice and humans (Fig. 4). In both species, individuals with a shorter chondrocranium have a higher braincase (upward shift of bregma) and a more inferior position of the posterior cranial vault/base. Individuals with a longer chondrocranium have a shorter braincase (downward shift of bregma), and especially in humans the skull is elongated in the antero–posterior axis (Fig. 6).

**Fig. 5:**
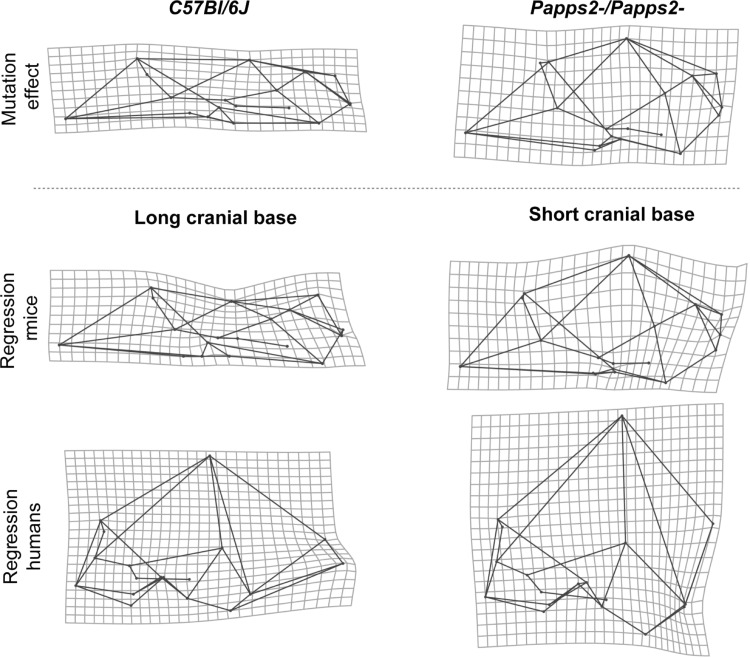
Morphological correspondence between the mutation effect on chondrocranial length and normal cranial shape variation in wildtype mice and humans. *Top row* average shape differences between *C57Bl/6J* wildtype mice and *Papps2*-*/Papps2*- mutant mice with decreased chondrocranial length (*left* shape changes from mutant to wildtype, *right* shape changes from wildtype to mutant). *Middle and bottom rows* TPS grids from multivariate regression of cranial shape on chondrocranial length representing the cranial shape associated with long and short chondrocraniums in *C57Bl/6J* mice and modern humans. Shape changes are exaggerated for visualization

Chondrocranial length is 9.2 % shorter in *Papps2*-/*Papps2*- mutant mice than in *C57Bl/6J* wildtype mice (*p* < 0.0001). The Procrustes distance between mutant and wildtype mice is 0.076 Procrustes units, and the amount of shape change predicted for a 9.2 % difference in chondrocranial length in *C57Bl/6J* wildtype mice corresponds to a Procrustes distance of 0.026. Visual comparison of the cranial phenotypic effect of the *Papps2*- mutation with normal skull variation associated with long and short braincases (Fig. 5) shows correspondence between shape patterns. Overall, *Papps2*-*/Papps2*- mutant mice present, as *C57Bl/6J* wildtype mice with short cranial bases, more rounded and expanded braincases, and more flexed cranial bases. This skull shape pattern is also similar to that in modern humans (Fig. 5), in which the increase of cranial base flexion due to contraction of the posterior cranial vault is more pronounced than in wildtype mice.

### Overall Skull Size

**Fig. 6:**
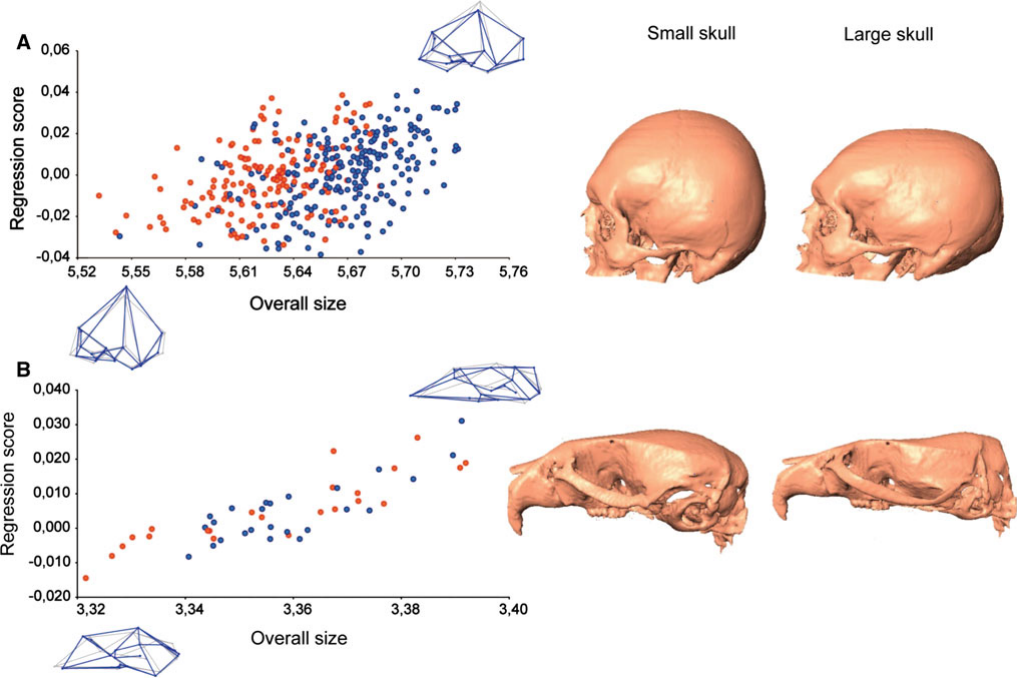
Multivariate regression of cranial shape on overall skull size in **a** modern humans and **b** wildtype *C57Bl/6J* mice. For more details see Fig. 2

The multivariate regression of hemicranial shape on overall size (Fig. 6) showed that in humans skull size explains a low but statistically significant percentage of total morphological variation (1.38 %; *p* < 0.001). In *C57Bl/6J* wildtype mice, the percentage of explained variation is higher (8.71 %; *p* < 0.001). The coefficient of variation for overall size in mice is 0.017 and 0.037 in humans. In wildtype mice and humans the skull shape changes follow a similar pattern in association with changes in overall skull size (Fig. 6). In both species, smaller skulls are associated with smaller faces and relatively larger and more rounded braincases, resulting in brachycephalic skulls. Individuals with large skulls are characterized by large prognathic faces with antero–posteriorly elongated and dorso–ventrally shortened (i.e. dolicocephalic) skulls. In larger individuals, the braincase is relatively smaller.

**Fig. 7:**
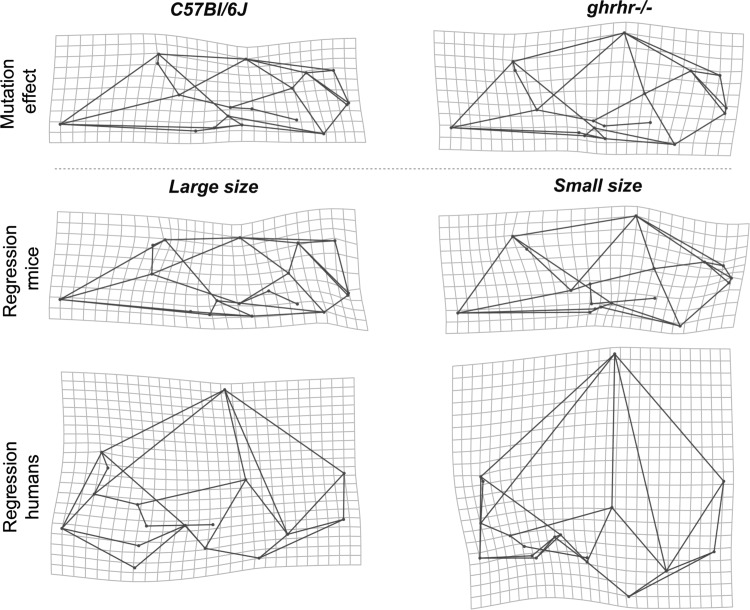
Morphological correspondence between the mutation effect on overall size and normal cranial shape variation in wildtype mice and humans. *Top row* average shape differences between *C57Bl/6J* wildtype mice and *ghrhr*-/- mutant mice with decreased overall size (*left* shape changes from mutant to wildtype, *right* shape changes from wildtype to mutant). *Middle and bottom rows* TPS grids from multivariate regression of cranial shape on overall size representing the cranial shape associated with large and small skulls in *C57Bl/6J* mice and modern humans. Shape changes are exaggerated for visualization

Statistical comparison of *ghrhr*-/- mutant mice and *C57Bl/6J* wildtype mice showed that skull size is 14.5 % smaller in *ghrhr*-/- mutant mice (*p* < 0.001). The cranial shape difference between *ghrhr*-/- and *C57Bl/6J* mice is 0.053 Procrustes units, which is almost the same as the predicted amount of cranial shape change corresponding to such an increase in overall size (0.057 Procrustes units). The average shape differences between *ghrhr*-/- mutant mice and *C57Bl/6J* wildtype mice consist of the typical allometric enlargement of the face relative to the neurocranium, and resemble the regression of shape on overall size in *C57Bl/6J* wildtype mice and humans (Fig. 7), following the same pattern as explained above.

## Discussion

We studied normal phenotypic variation of cranial shape in a mixed population of inbred wildtype mice and in one human population, and compared it to the phenotypic effects of three different genetic mutations using corresponding mouse models. For the three mutations, their phenotypic effects were mirrored by a similar pattern of variation in humans that explained a statistically significant proportion of the total variation in craniofacial shape. Thus, the effects of mutations on brain size, chondrocranial size and overall cranial size corresponded to sets of correlated changes or axes of covariation in human cranial shape. We argue below that these axes of covariation likely reflect, at a high level, similar developmental causes even though the specific genetic and developmental determinants of variation along them are likely to be complex and highly varied. In effect, we argue that these patterns reveal the existence of key developmental processes that organize the expression of phenotypic variation in the vertebrate skull.

Our study has several limitations that impinge on the interpretation of the results reported here. The first, and most obvious, is that the mouse mutants studied here, which express fairly extreme phenotypes, do not reflect the complexity of genetic variation that is subject to natural selection. Similarly, the wildtype controls are homozygous inbred mice and so exhibit an unusual pattern of variation compared to natural populations. Most neutral as well as adaptive evolutionary processes are based on relatively continuous phenotypic variation within a population. The genetic basis of such variation is usually not attributable to allelic variation of a single gene, and the evolution of complex phenotypes typically involves changes in multiple genes, some of which may partly compensate the effect of others (Pavlicev and Wagner 2012). On the other hand, the genetic and developmental system can evolve even though the average population phenotype stays the same (Muller and Wagner 1996; True and Haag 2001; Rice 2002). It is highly unlikely, for these reasons among others, that the relative growth of the chondrocranium in mammals is frequently altered via modulation of the PAPPS2 pathway in nature because there are very many other developmental-genetic ways in which that can happen, some of which may not have the deleterious effects of the PAPPS2 null mutation (structural effects in cartilage that predispose osteoarthritis (Ford-Hutchinson et al. 2007).

Secondly, these mutants represent singular instances in which the hypothesized developmental process is perturbed. Increasing brain size may produce somewhat different phenotypic effects if it occurs via a different mechanism that may, for example act differently over the ontogenetic trajectory or produce region-specific effects on the brain. Further studies in which the same hypothetical process is perturbed in multiple ways are necessary to flesh out the extent of this kind of variation.

The third and most significant limitation is that the mutations used here vary in the degree to which they are fully understood and this impacts our confidence in the inference that the differences between each mutant and the wildtype is due to a perturbation of each hypothesized developmental process. This issue is best addressed individually for each mutant. For example, the *Mceph* mutation, an 11 bp deletion in the Kcna1 gene (Diez et al. 2003; Petersson et al. 2003), exhibits an overgrowth of the brain due to neural cell hypertrophy that occurs most markedly in the hippocampus, parietal cortex and ventral cortex. The overgrowth is marked by 3 weeks but continues to 12 weeks, which is well beyond the period of normal brain growth. Finally, the *Mceph* mutation also appears to have effects related to the IGF pathway, which may account for the slightly reduced body mass seen in these mice (Petersson et al. 1999). This model thus departs from what one might expect in evolutionary changes in brain growth in the timing of growth and, to a lesser degree, its anatomical localization. These differences between the *Mceph/Mceph* model and what we expect is happening during evolutionary changes in brain size would be expected to reduce the similarity between the shape effects of the *Mceph* mutation and the brain size—cranial shape regressions in both mice and humans. The fact that we still see similar changes in both cases to what we observe in the *Mceph* mutants supports our inference that these axes of covariation reflect underlying variation in brain size.

In the case of the brachymorph (*Papps2*-*/Papps2*-) mouse, there is good evidence that cartilage growth is perturbed. In particular, the growth plates including synchondroses are affected in that they appear disorganized with reduced rates of maturation through the growth plate resulting in slower growth (Vanky et al. 2000). Thus, the growth rates of the cranial synchondroses are reduced in these mice. It is not known, however, to what extent and how other aspects of cartilage growth, such as appositional growth of the condylar cartilage, are affected by the mutation. Interestingly, achondroplastic mice carrying mutations in fibroblast growth factor receptors exhibit cranial morphology that is remarkably similar to the brachymorph mice based on qualitative description (Jolly and Moore 1975; Rice et al. 2003). This suggests that a generalized reduction on cartilage growth may occur in brachymorph mice. Here, we were interested specifically in the effect of the mutation on the growth of the chondrocranium. The length of the cranial base is determined by chondrocyte proliferation and maturation within the cranial synchondroses and we know that this dimension is significantly reduced in brachymorph mice. This was the variable that we regressed cranial shape on for the mouse and human samples. The comparison of those regressions to the brachymorph mice is thus valid but somewhat complicated by the possibility that other aspects of cranial shape may also be affected as a result of the brachymorph mutation.

Finally, even the model that we used to investigate the effects of generalized reduction in growth is not entirely straightforward. Little mice exhibit reduced post-natal growth but normal growth in utero. This is also true for humans born with mutations in the homologous gene (Alba and Salvatori 2004). This is despite the fact that the growth hormone pathway does influence size throughout ontogeny (Waters and Kaye 2002). This illustrates the complexity of the pathways that regulate somatic growth in mammals and calls in question the extent to which this seemingly simple parameter can be thought of as one underlying process. Genome wide associations studies demonstrate the genetics of human stature are extraordinarily complex and that this trait is likely influenced by very many loci in diverse pathways, most of which are likely common and each of which has small effects (Weedon et al. 2008; Yang et al. 2010; Zhao et al. 2010; Lanktree et al. 2011). It is not known to what extent modulation of the growth hormone pathway underlies evolutionary changes in body size. Given the genetic complexity of stature in humans, however, there is no reason to expect this pathway, important as it is for determining individual growth, to be a key driver of evolutionary change in body size. This limitation must be considered when interpreting the relevance of mouse models like the *ghrhr*-/- mouse model for evolution. Further, the fact that mice and humans are at opposite extremes of the range of mammalian body sizes complicates interpretation of the *ghrhr*-/- mouse model. As John Bertram (1988) has shown, for example, the functional implications of size variation (for jaw mechanics, for example) can vary dramatically across such large size ranges.

Given these caveats, the results of our analysis of these three developmental determinants of overall craniofacial shape are all the more striking. We show here that homologous axes of covariation exist in mice and in humans that are likely related to key gross-level developmental processes such as brain growth, chondrocranial growth and overall body size. Developmental factors such as these have previously been proposed as large-scale determinants of craniofacial variation. DeBeer made arguments like this when discussing the similarity of achondroplastic forms of multiple mammals or of the apparent effects of growth and scaling (DeBeer 1937) and this kind of argument is implicit in the functional matrix model of Moss (Moss and Young 1960; Moss 1962; Moss and Salentijn 1969) or in Enlow’s deconstruction of cranial development (Enlow and Azuma 1975; Enlow 1990). Implicit in these arguments is the idea that the vast potential for variation in developmental pathways is somehow “funneled” into a few key processes that are the major determinants of cranial morphology (Hallgrímsson et al. 2007a; Hallgrímsson and Lieberman 2008; Lieberman et al. 2008).

In this paper we tried to study the structure and complexity of cranial development and the resulting variational properties at the phenotypic level. We found that the effects of the three predictor variables brain size, chondrocranial length, and overall size on cranial shape are qualitatively similar among mice and humans. To some degree they also reflect the cranial shape changes induced by mutations affecting these properties.

Despite the similarity of shape patterns, we found differences in the variational properties. The coefficients of variation of the three predictor variables were clearly larger in humans than in mice, suggesting a more complex developmental system in humans composed of a larger number of genetic and developmental factors. This interpretation is also supported by the generally lower fractions of shape variance explained by these factors in humans as compared to mice.

We further compared the average amount of cranial shape difference between mutant mice and the corresponding wildtype population (Procrustes distance between the mean shapes) to the amount of shape change predicted for the difference in the three predictor variables using multivariate regressions in wildtype populations. Except for overall cranial size, this predicted shape difference was much less than the amount of shape difference observed for the mutations. Assuming that the mutations act mainly via a single developmental pathway on cranial shape, this result indicates that the regressions are a summary of multiple pathways, some of which are not directly causal (e.g., factors influencing both brain size and cranial shape) or have contradictory effects that partly cancel on average. Despite the apparent funneling of developmental processes by factors such as brain size, or chondrocranial length, the effects of these variables on cranial shape do appear to be complex and not well represented by a single linear component. This is likely the reason for the generally low fractions of explained cranial shape variance by the regressions both in mice and humans.

Although complex, these findings are important because mouse models are commonly used to assess vertebrate and also primate development. They are used for studying human cranial deformations and teratologies. It is reassuring to know that the signatures of the same developmental interactions can be detected in both mice and humans despite the large differences in craniofacial shape and function. More importantly, it is also reassuring that these hypothesized processes account for significant proportions of overall cranial variation in humans and mice. Although the percentages of total variance explained here leave much room for other factors, they are large compared to the underlying genetic complexity of these traits. Gene effects larger than 1 % of the phenotypic variance are rare in GWAS studies (McCarthy et al. 2008) but these axes of covariation explain from 1 to 9 % of the total phenotypic variance which is significant given the underlying developmental complexity of the craniofacial complex.

The findings of this study contribute to our understanding of how development relates to the evolution of the craniofacial complex. Natural selection acts on the phenotype, and phenotypic variation is structured by developmental processes. It is this structuring of variation in the form of integration and canalization that makes development relevant to evolutionary explanations. Two of us have argued previously, therefore, that unraveling the developmental determinants of evolvability is the central question of evolutionary developmental biology (Hendrikse et al. 2007). We have shown here that in both mice and humans variation is structured by similar developmental processes that contribute significantly to phenotypic variation. It is likely, therefore, that these processes are targets of selection for cranial morphology. As such they both enable and constrain the morphological variation that is produced.
